# Evaluating the Anti-Melanoma Effects and Toxicity of Cinnamaldehyde Analogues

**DOI:** 10.3390/molecules28217309

**Published:** 2023-10-28

**Authors:** Rongsong Jiang, Fukui Shen, Miaomiao Zhang, Shulipan Mulati, Jinfeng Wang, Yicun Tao, Weiyi Zhang

**Affiliations:** 1 School of Pharmacy, Xinjiang Medical University, Urumchi 830017, China; jrs@stu.xjmu.edu.cn (R.J.); zmm@stu.xjmu.edu.cn (M.Z.); slp@stu.xjmu.edu.cn (S.M.); wjf@xjmu.edu.cn (J.W.); 2 State Key Laboratory of Medicinal Chemical Biology, College of Pharmacy, Nankai University, Tianjin 300353, China; fukuishen@126.com

**Keywords:** cinnamaldehyde analogues, anti-melanoma, ENO1, apoptosis, p38 pathway

## Abstract

Cinnamaldehyde (CA) showed potent activity against melanoma in our previous study, and the structure of unsaturated aldehydes is envisaged to play a role. Nevertheless, its limited drug availability restricts its clinical application. Therefore, a series of CA analogues were synthesized to evaluate their anti-melanoma activities across various melanoma cell lines. These compounds were also tested for their toxicity against the different normal cell lines. The compound with the most potential, CAD-14, exhibited potent activity against the A375, A875 and SK-MEL-1 cells, with IC_50_ values of 0.58, 0.65, and 0.82 µM, respectively. A preliminary molecular mechanism study of CAD-14 indicated that it could inhibit the p38 pathway to induce apoptosis, and suppress tumor growth by inhibiting the expression of ENO1. Furthermore, an acute toxicity study depicted that CAD-14 has better safety and tolerability than CA in vivo. These findings indicate that CAD-14 might be a lead compound for exploring effective anti-melanoma drugs.

## 1. Introduction

Recently, covalent inhibitors have attracted increasing attention as an important complement and alternative to non-covalent counterparts. Natural products have emerged as a fundamental resource for the development of covalent drugs [1]. As the potential targets of nucleophilic residues, activated cysteine and serine residues are expected to be the most-attacked binding site by Michael acceptors, aziridines, epoxides, and β-lactams [2,3,4]. While early covalent inhibitors relied heavily natural products, the design of contemporary targeted covalent inhibitors has also necessitated an element of serendipity in their development [5].

Melanoma originates in melanin cells in the skin, mucous membrane, and uveitis. Specifically, three types of genes are involved in melanoma genetic risk: those controlling telomerase, those controlling cell cycling and proliferation, and those controlling tumor–immunity interactions [6]. The disease’s high degree of malignancy, high metastasis, and poor prognosis are the major causes of skin cancer-related deaths [7]. Malignant melanoma can regulate the local environment and body homeostasis through various mechanisms [8]. Based on epidemiological data statistics, the global incidence rate of malignant melanoma is experiencing an annual increase of 3–5%, and 130,000 new cases of melanoma and 50,000 melanoma-related deaths are diagnosed yearly, making it one of the fastest-growing malignant tumors worldwide [9]. The traditional treatment means are surgery, radiotherapy, and chemotherapy, but the five-year survival rate for patients with metastatic melanoma stands at a mere 27% due to the prevalence of adverse drug reactions and significant drug resistance [10]. There has been growing acknowledgment of the widespread presence of v-rafmurine sarcoma viral oncogene homolog B (BRAF) mutational heterogeneity in melanoma within academic circles. Vemurafenib was the first to show efficacy in BRAF-mutant melanoma followed by dabrafenib. Although immunotherapy and targeted therapy for BRAF have significantly improved the overall survival and quality of life of patients with advanced melanoma, some patients still do not respond to them [11]. Consequently, the development of novel drugs and the identification of their underlying mechanisms are of paramount importance.

Cinnamaldehyde (CA), a prominent natural product, serves as the main component of the volatile oil derived from cinnamon and various Cinnamomum species [12]. Recent studies have depicted its enormous potential as a novel resource for research and development in the creation of anti-tumor medications. Our previous works have demonstrated that CA improves this anti-melanoma effect, and the structure of unsaturated aldehydes is envisaged to play a role [13]. However, due to its limited drug availability, it is unsuitable for clinical use. This study aims to design and synthesize a series of CA analogues while retaining the α, β-unsaturated aldehyde moiety. We achieved the best activity from CAD-14 analogues during preliminary screening. Additionally, a preliminary molecular mechanism study of CAD-14 indicated that it could inhibit the p38 pathway to induce apoptosis. Meanwhile, CAD-14 inhibited tumor growth by inhibiting ENO1 in vivo. Furthermore, an acute toxicity study demonstrated that CAD-14 has superior safety and tolerability compared with CA. This study will establish a new theoretical foundation for CAD-14 as a potential anti-tumor drug.

## 2. Results

### 2.1. Structure Elucidation of CA Analogues

**Scheme 1:**
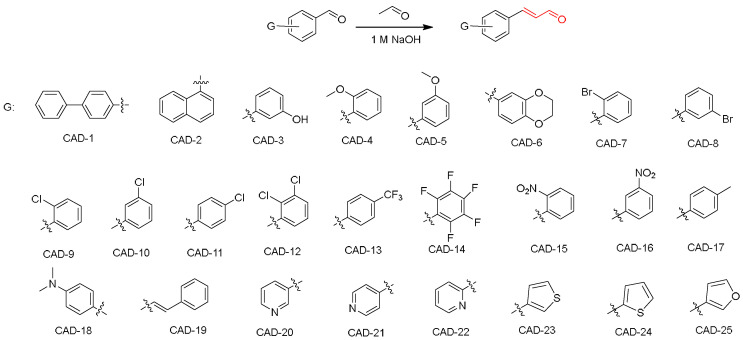
The synthesis of compounds CAD **1**–**25**. H_2_O/THF; 0 °C–25 °C; 21.1–88.1% yield in this step.

**Figure 1:**
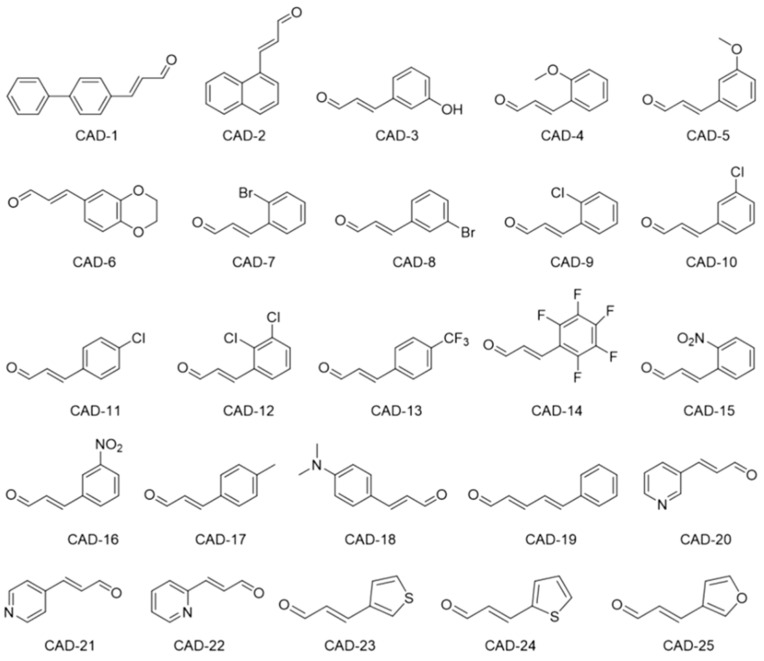
The structures of CA analogues.

Using CA as a basis, we retained its enal structure, then constructed a molecular skeleton and added selective groups using a drug molecular heterozygous design strategy. The electrical modification and steric hindrance substitution of a CA benzene ring were performed first, following the principle of least modification; this approach can enhance the activity and selectivity of the drug, but it may also significantly change the drug activity. In addition, the structure of the parent nucleus may also affect the drug’s pharmacodynamic activity. Accordingly, we modified the benzene ring of the parent nucleus to introduce lipophilic furan ring and thiophene ring compounds. Analogues with biphenyl, naphthalene, and 1,4-dioxane were prepared as substitutes for the benzene ring of CA, to explore the impact of different parent structures on the compound’s pharmacological properties. The chemical structures are illustrated in Scheme 1 and Figure 1.

### 2.2. Evaluating the Anti-Melanoma Activity of Synthesized Analogues

**Figure 2:**
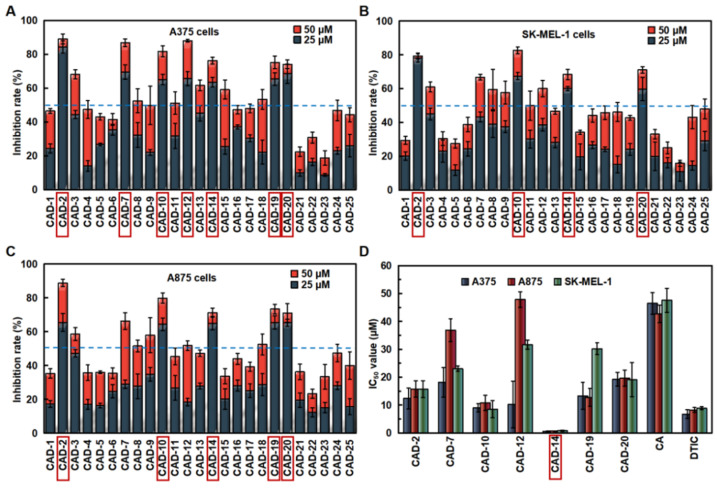
Effect of CA analogues in melanoma cells. (**A**–**D**) The different melanoma cell lines were treated with 0–50 µM analogues for 72 h, and then IC_50_ value was assayed (A dashed blue line: 50% inhibition rate; red box: analogues with high activity).

The initial screening of the synthesized analogues against various phenotypes’ melanoma cell lines (A375, A875, SK-MEL-1) revealed that analogues CAD-2, CAD-7, CAD-10, CAD-12, CAD-14, CAD-19, and CAD-20 had strong activity (Figure 2A–C). The IC_50_ values of CAD-14 were 0.58, 0.65, and 0.82 μM in A375, A875, and SK-MEL-1 cell lines, respectively. These findings display that CAD-14 had the best effect, which was 80 times stronger than that of CA. Compared with the positive drug DTIC, the effect was nearly 15 times stronger (Figure 2D). These results suggest that CAD-14 may have stronger anti-melanoma activity compared to CA.

### 2.3. CAD-14 Inhibited the Growth of A375 Cells and Arrested Cell Cycle

**Figure 3:**
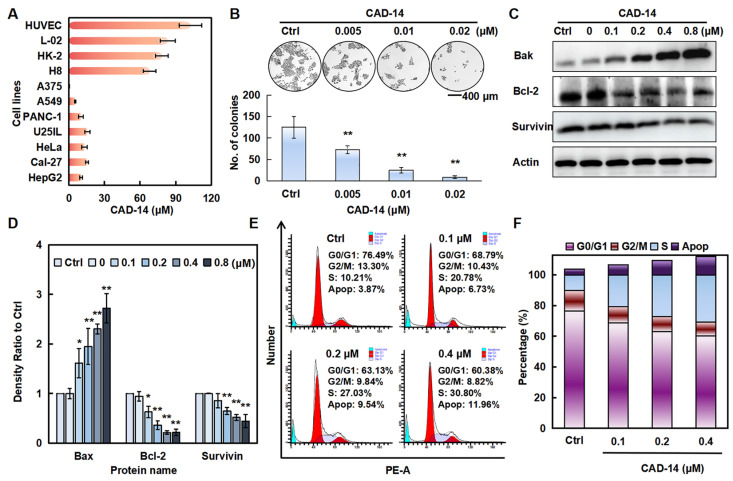
CAD-14 inhibited the proliferation of A375 cells. (**A**) The different tumor and normal cell lines were treated with 0–200 µM CAD-14 for 72 h, and then IC_50_ value was assayed. (**B**) Cells were treated with 0–0.02 µM CAD-14 for 14 days, and Giemsa staining was used to conduct colony formation assays; the histogram depicts the number of colonies. (**C**,**D**) Cells were treated with 0–0.8 µM CAD-14 for 24 h; then, the related proteins were tested via Western blotting. Histograms depict the intensity ratio. (**E**,**F**) Cells were treated with 0–0.4 µM CAD-14 for 24 h, then the DNA content of the cells was determined with a flow cytometry system. Histograms show the percentage of cells in G0/G1, G2/M, and S phase. Values represent the means ± SD. * *p* < 0.05, ** *p* < 0.01 versus “Ctrl” group.

To evaluate the specificity of CAD-14 towards various cell types, multiple types of cancer cells and normal cells were tested. CAD-14 inhibited A375 cells more effectively than other cancer cells and was less sensitive to normal cell lines (Figure 3A). A375 cells were chosen for the follow-up experiments. A colony-forming assay was used to establish that CAD-14 exhibits proliferation inhibitory activity. Compared to the “Ctrl” group, CAD-14 significantly reduced the number of colonies (Figure 3B). To determine whether the cytotoxic effect of CAD-14 was associated with apoptosis, we tested the apoptosis-related protein via Western blotting. As depicted in Figure 3C,D and Supplement Figure S1, the expression of the pro-apoptotic protein Bak was strongly increased, while the expression of the anti-apoptotic proteins Bcl-2 and survivin was substantially diminished after treatment with CAD-14 in a dose-dependent manner. In order to investigate whether cell cycle arrest contributed to CAD-14-induced cell growth inhibition, the cell cycle distribution was analyzed using a flow cytometer. As displayed in Figure 3E,F, CAD-14 markedly induced S phase arrest in A375 cells in a concentration-dependent manner. These findings suggest that CAD-14 might substantially inhibit the proliferation of A375 cells and arrest the S phase.

### 2.4. CAD-14 Induced Apoptosis and Inhibited the P38 Pathway

**Figure 4:**
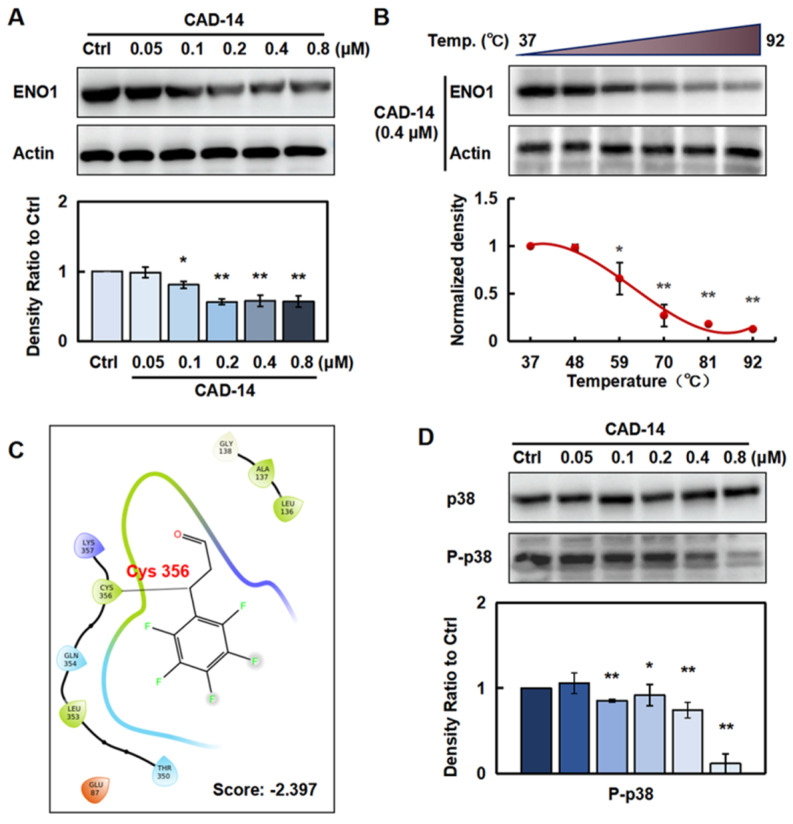
Validation of the CAD-14 anti-melanoma pathway. (**A**) Cells were treated with 0–0.8 µM CAD-14 for 24 h, and the ENO1 was assayed via Western blotting. (**B**) CAD-14 treatment (0.4 μM) decreased the thermal stability of ENO1 in cell lysates, as measured using a temperature-dependent CETSA. (**C**) Molecular docking analysis of ENO1 and CAD-14. (**D**) Cells were treated with 0–0.8 µM CAD-14 for 24 h, and the p-38/P-p38 were assayed via Western blot. Histograms show the intensity ratio. Values represent the means ± SD. * *p* < 0.05, ** *p* < 0.01 versus “Ctrl” group.

To evaluate the effect of CAD-14 on ENO1, Western blot and a CETSA assay were then performed. The results indicated that CAD-14 changed the stability of ENO1 protein in a temperature- and dose-responsive manner (Figure 4A,B and Supplementary Figures S2 and S3). To predict the potential position of CAD-14 binding on ENO1, multiple molecular dynamics simulations of ENO1 (PDB ID: 3B97) complexed with CAD-14 and other analogues were conducted. Molecular docking analysis revealed that CAD-14 might covalently bind to ENO1 at Cys356 via a Michael addition reaction (Figure 4C and Supplementary Table S1). Research has found that the p38 signaling pathway plays a crucial role in the occurrence/development of melanoma and has become one of the important targets for its clinical treatment [14]. Therefore, we detected the expression of p38/P-p38. The results depict that CAD-14 suppressed the phosphorylation of p38 but did not affect its total protein levels (Figure 4D and Supplementary Figure S4). Accordingly, we propose that CAD-14 inhibited the p38 pathway to induce cell apoptosis. 

### 2.5. CAD-14 Inhibited Tumor Growth by Inhibiting ENO1 In Vivo

**Figure 5:**
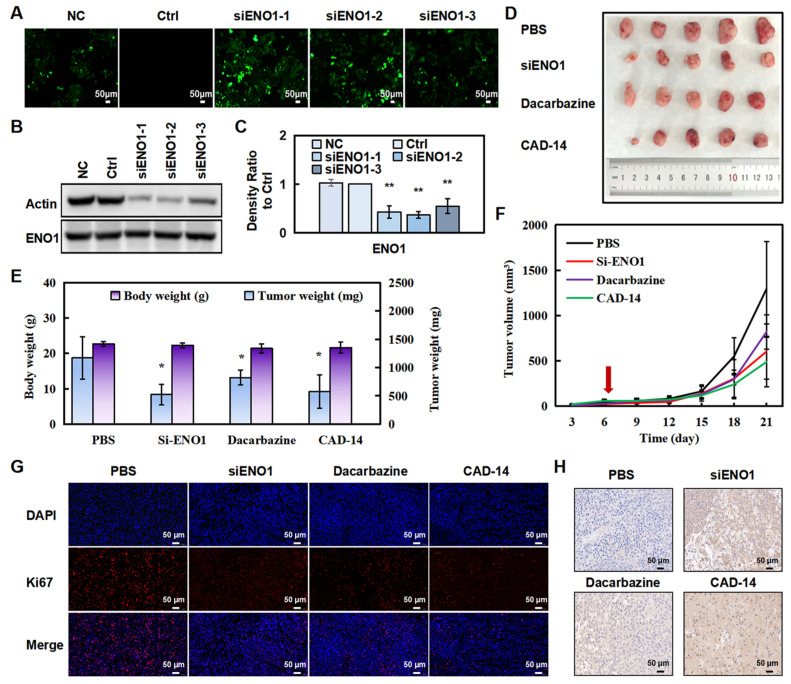
CAD-14 inhibited the growth of A375 cells in vivo. (**A**–**C**) The A375 cells were transfected with three different recombinant ENO1 retroviral vectors and an NC virus for 72 h; the effect of recombinant retrovirus on ENO1 expression were evaluated via fluorescence photography and Western blotting. Histograms show the intensity ratio. (n = 3). Values represent the means ± SD. ** *p* < 0.01 versus “NC” group. (**D**–**F**) Cells were injected subcutaneously into nude mice. Then, they were treated with Dacarbazine (10 mg/kg/day, as a positive control) or CAD-14 (5 mg/kg/day) for two weeks (the red arrow at day 7 represents the first day of treatment). The tumor volume was calculated (n = 5). (**G**,**H**) Immunofluorescent staining of Ki67 and ENO1 to detect proliferation ability and expression of ENO1 in tumor tissues, respectively. Values represent the means ± SD. * *p* < 0.05 versus “PBS” group.

To further evaluated whether ENO1 is a target protein of CAD-14, we next knock-out the ENO1 gene in A375 cells; Figure 5A–C and Supplementary Figure S5 showed the efficiencies of three different recombinant ENO1 retroviral vectors. Based on these results, we finally selected “siENO1-2” for next animal experiments. The results showed that both “siENO1” and “CAD-14” groups can inhibit the tumor growth in the xenograft model compared with the “PBS” group (Figure 5D–F), and the effect was better than dacarbazine. Besides this, we performed immunofluorescence staining on Ki67 (one proliferation-related marker). There was obvious decrease in Ki67-positive cells in “CA” groups (Figure 5G). Meanwhile, we detected the expression of ENO1 and found that there were fewer ENO1 accumulated in the “siENO1” and “CAD-14” groups (Figure 5H). Taken together, these findings suggest that CAD-14 substantially inhibits the tumor growth via knock-out ENO1 in vivo.

### 2.6. Acute Toxicity Study of CAD-14 In Vivo

**Figure 6:**
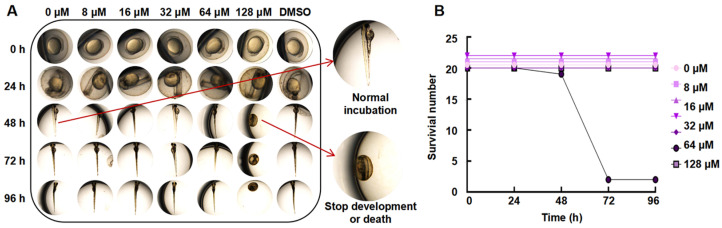
Developmental effects of CAD-14 on embryos. (**A**) The embryo morphology after CAD-14 treatment for 0–96 h. (**B**) Survival statistics in different time and concentration.

To evaluate the toxicity of CAD-14 during embryonic development, an acute toxicity assay was performed using *zebrafish* embryos. As displayed in Figure 6, Supplementary Figure S12 and Table 1, death and malformation began to occur simultaneously after CAD-14 (128 μM) exposure for 96 h, with mortality rates of 90.00%. Furthermore, the median death concentration (IC_50_) was 83.68 μM. These results indicate that CAD-14 might have toxic and teratogenic effects after long-term treatment with high concentrations.

## 3. Discussion

α-β-unsaturated carbonyl groups are the most common and potentially active substructures in natural products. One sixth of all known natural products contain this chemical signature. However, there is controversy as to whether these compounds can be used as leads for drug design, because their potential Michael receptor function may lead to side effects such as cell damage or cytotoxicity [14]. Simultaneously, α, β unsaturated carbonyl groups possess the capability to function as free-radical scavengers, facilitating the covalent binding of ligands to specific proteins, while also exhibiting antioxidant properties. Through α, β unsaturated carbonyl groups can interact directly with enzymes to activate certain enzymes, thereby preventing carcinogenesis. For example, quinone reductases and glutathione S-transferase activities can be activated, and subsequently inhibit the electrophilic reaction of carcinogens [15]. These effects can be attributed to the properties of the Michael receptor.

Essentially, CA exhibits two distinct reaction pathways: one is the Michael addition reaction, wherein the thiol group of cysteine reacts with the double bond of α-β-unsaturated aldehyde-ketone; the other is the formation of imines, facilitated by an abnormally electrophilic aldehyde group [16]. The literature has reported that CA can be covalently modified with the reactive cysteine of TRPA1, resulting in a Michael addition reaction, which activates signal transduction pathways and rapidly transmits potential tissue damage signals through the pain pathway [17]. Most of the molecular targets of CA causing cellular effects are unknown. 

ENO1 is expressed on the cell surface, can promote cancer invasion, and is subject to a series of specific post-translational modifications, such as acetylation, methylation, and phosphorylation [18]. In tumor tissues, overexpressed ENO1 may be released into the peripheral blood through apoptosis of tumor cells or other pathways, thereby leading to up-regulation of ENO1 protein expression in the peripheral blood of tumor patients [19]. In addition, ENO1 expression also affects the cell cycle. Studies have demonstrated that when the ENO1 gene is silenced by siRNA, the proliferation of HCC cells is inhibited, the S phase of the cell cycle is shortened, and the G2/M phase is prolonged [20]. In this study, we designed a series of CA analogues through further structural optimization of CA. Among them, CAD-14 has strong anti-tumor effects because it is added the electron-withdrawing groups, increasing the electrophilicity of the aldehyde. Besides this, CAD-14 may participate in the Michael addition reaction with ENO1 through α and β-unsaturated aldehydes and ketones. This covalent binding changes the stability of ENO1, leads to S cell cycle arrest, and triggers apoptosis by inhibiting the p38 pathway. Meanwhile, CAD-14 may inhibit tumor growth by inhibiting ENO1 in vivo. This provides a more powerful basis for the study of melanoma treatment. Furthermore, this study also demonstrates that α-β-unsaturated aldehydes play a key role in anti-melanoma effects.

Recent pharmacokinetic and safety studies have revealed that in the acute poisoning of rats after intravenous injection of CA, the lethal dose (LD_50_) value was 74.8 mg/kg [21]. In addition, when the dose of CA reached a certain degree, the micronucleus rate in hepatocytes was increased (rats: 1100 mg/kg, mice: 850–1700 mg/kg). These results indicate that high doses of CA can cause genetic changes in hepatocytes [22]. *Zebrafish* embryos are commonly used as animal models for testing drug toxicity. This study indicated that CAD-14 is safe at normal concentrations and could be used as a potential anti-cancer drug.

## 4. Materials and Methods

### 4.1. Chemistry (Procedure for the Synthesis of Compounds ***1**–**25***)

Preparation of (*E*)-3-([1,1′-biphenyl]-4-yl)acrylaldehyde (CAD-1) [23]: Under an argon atmosphere, [1,1′-biphenyl]-4- carbaldehyde (182 mg, 1 mmol) was dissolved in 3 mL tetrahydrofuran under ice bath, 40% acetaldehyde aqueous solution (170 μL, 1.2 mmol) and 1 mL 1 mol/L NaOH aqueous solution were added to react for 3 h. The crude products were purified by column chromatography (petroleum ether: ethyl acetate = 6:1). The product compound 1 was obtained as a pale yellow solid, 173.4 mg, with a yield of 83.1%. ^1^H NMR (400 MHz, CDCl_3_) *δ* 9.82 (d, *J* = 7.5 Hz, 1H), 7.64 (s, 4H), 7.68–7.56 (m, 2H), 7.48–7.29 (m, 4H), 6.74 (dd, *J* = 16.1, 8.1 Hz, 1H). MS (EI) *m/z*: 208, found 208.
Preparation of (*E*)-3-(naphthalen-1-yl)acrylaldehyde (CAD-2) [24]: Under an argon atmosphere, 1-naphthaldehyde (156 mg, 1 mmol) was dissolved in 3 mL tetrahydrofuran under ice bath; 40% acetaldehyde aqueous solution (170 μL, 1.2 mmol) and 1 mL 1 mol/L NaOH aqueous solution were added to react for 8 h. The crude products were purified via column chromatography (petroleum ether: ethyl acetate = 6:1). The product compound 2 was obtained as a pale yellow solid, 60.5 mg, with a yield of 33.2%. ^1^H NMR (400 MHz, CDCl_3_) *δ* 9.62 (dd, *J* = 6.1, 1.0 Hz, 1H), 8.03–7.79 (m, 3H), 7.66 (t, *J* = 7.5 Hz, 1H), 7.62–7.53 (m, 3H), 7.52–7.43 (m, 1H), 6.58 (dd, *J* = 15.1, 6.1 Hz, 1H). MS (EI) *m/z*: 182, found 182.
Preparation of (*E*)-3-(3-hydroxyphenyl)acrylaldehyde (CAD-3) [25]: Under an argon atmosphere, 4-methylbenzaldehyde (120 mg, 1 mmol) was dissolved in 3 mL tetrahydrofuran in an ice bath; 40% acetaldehyde aqueous solution (170 uL, 1.2 mmol) and 1 mL 1 mol/L NaOH aqueous solution were added to react for 4 h. The crude products were purified via column chromatography (dichloromethane: methanol = 10:1). The product compound 3 was obtained as a pale yellow oil, 77 mg, with a yield of 52.0%. ^1^H NMR (400 MHz, CDCl_3_) *δ* 9.62 (dd, *J* = 8.4, 2.4 Hz, 1H), 7.44 (d, *J* = 15.9 Hz, 1H), 7.08 (d, *J* = 7.8 Hz, 1H), 6.98 (t, *J* = 5.0 Hz, 3H), 6.84 (d, *J* = 8.8 Hz, 1H), 6.28 (dd, *J* = 15.0, 8.1 Hz, 1H). MS (EI) *m/z*: 148, found 148.
Preparation of (*E*)-3-(2-methoxyphenyl)acrylaldehyde (CAD-4) [23]: Under an argon atmosphere, 2-methoxybenzaldehyde (136 mg, 1 mmol) was dissolved in 3 mL tetrahydrofuran in an ice bath; 40% acetaldehyde aqueous solution (170 uL, 1.2 mmol) and 1 mL 1 mol/L NaOH aqueous solution were added to react for 4 h. The crude products were purified via column chromatography (petroleum ether: ethyl acetate = 6:1). The product compound 4 was obtained as a pale yellow solid, 114 mg, with a yield of 70.5%. ^1^H NMR (400 MHz, CDCl_3_) *δ* 9.70 (d, *J* = 7.9 Hz, 1H), 7.85 (d, *J* = 16.1 Hz, 1H), 7.56 (dd, *J* = 7.7, 1.7 Hz, 1H), 7.42 (ddd, *J* = 8.4, 7.4, 1.7 Hz, 1H), 7.01 (td, *J* = 7.6, 1.1 Hz, 1H), 6.96 (dd, *J* = 8.4, 1.1 Hz, 1H), 6.80 (dd, *J* = 16.1, 7.9 Hz, 1H), 3.92 (s, 3H). ^13^C NMR (100 MHz, Chloroform-d) *δ* 194.70, 158.30, 148.30, 132.73, 129.11, 128.90, 122.96, 120.90, 111.28, 55.59. MS (EI) *m/z*: 162, found 162.
Preparation of (*E*)-3-(3-methoxyphenyl)acrylaldehyde (CAD-5) [26]: Under an argon atmosphere, 3-methoxybenzaldehyde (136 mg, 1 mmol) was dissolved in 3 mL tetrahydrofuran in an ice bath; 40% acetaldehyde aqueous solution (170 uL, 1.2 mmol) and 1 mL 1 mol/L NaOH aqueous solution were added to react for 5 h. The crude products were purified via column chromatography (petroleum ether: ethyl acetate = 6:1). The product compound 5 was obtained as a pale yellow solid, 108 mg, with a yield of 66.7%. ^1^H NMR (400 MHz, CDCl_3_) *δ* 9.60 (dd, *J* = 6.2, 0.9 Hz, 1H), 7.43 (t, *J* = 7.5 Hz, 1H), 7.38 (dd, *J* = 15.0, 1.1 Hz, 1H), 7.24 (dd, *J* = 7.5, 1.0 Hz, 1H), 7.17 (q, *J* = 1.9 Hz, 1H), 7.01 (d, *J* = 7.5 Hz, 1H), 6.62 (dd, *J* = 15.2, 6.2 Hz, 1H), 3.83 (s, 3H). MS (EI) *m/z*: 162, found 162.
Preparation of (*E*)-3-(2,3-dihydrobenzo[b][1,4)dioxin-6-yl)acrylaldehyde (CAD-6) [27]: Under an argon atmosphere, thiophene-3-carbaldehyde (164 mg, 1 mmol) was dissolved in 3 mL tetrahydrofuran in an ice bath; 40% acetaldehyde aqueous solution (170 μL, 1.2 mmol) and 1 mL 1 mol/L NaOH aqueous solution were added to react for 5 h. The crude products were purified via column chromatography (petroleum ether: ethyl acetate = 6:1). The product compound 6 was obtained as a pale yellow solid, 125.4 mg, with a yield of 66.1%. ^1^H NMR (400 MHz, CDCl_3_) *δ* 9.83 (d, *J* = 2.1 Hz, 1H), 7.41 (p, *J* = 2.6, 1.9 Hz, 2H), 7.09 (d, *J* = 10.2 Hz, 1H), 6.99 (dd, *J* = 8.6, 2.2 Hz, 1H), 6.91 (d, *J* = 8.6 Hz, 1H), 4.33–4.27 (m, 4H). MS (EI) *m/z*: 190, found 190.
Preparation of (*E*)-3-(2-bromophenyl)acrylaldehyde (CAD-7) [23]: Under an argon atmosphere, 2-bromobenzaldehyde (184 mg, 1 mmol) was dissolved in 3 mL tetrahydrofuran under ice bath; 40% acetaldehyde aqueous solution (170 μL, 1.2 mmol) and 1 mL 1 mol/L NaOH aqueous solution were added to react for 3 h. The crude products were purified via column chromatography (dichloromethane: methanol = 10:1). The product compound 7 was obtained as a pale yellow oil, 184.5 mg, with a yield of 88%. ^1^H NMR (400 MHz, CDCl_3_) δ 9.73 (d, *J* = 7.6 Hz, 1H), 7.87–7.47 (m, 2H), 7.45–7.34 (m, 2H), 7.27 (ddd, *J* = 7.5, 6.1, 3.4 Hz, 1H), 6.46 (dd, *J* = 15.0, 6.2 Hz, 1H). MS (EI) *m/z*: 210, found 210.
Preparation of (*E*)-3-(3-bromophenyl)acrylaldehyde (CAD-8) [28]: Under an argon atmosphere, 3-bromobenzaldehyde (184 mg, 1 mmol) was dissolved in 3 mL tetrahydrofuran in an ice bath; 40% acetaldehyde aqueous solution (170 μL, 1.2 mmol) and 1 mL 1 mol/L NaOH aqueous solution were added to react for 6 h. The crude products were purified via column chromatography (petroleum ether: ethyl acetate = 6:1). The product compound 8 was obtained as a pale yellow solid, 120.1 mg, with a yield of 57.2%. ^1^H NMR (400 MHz, CDCl_3_) *δ* 9.71 (d, *J* = 7.6 Hz, 1H), 7.71 (t, *J* = 1.8 Hz, 1H), 7.57 (ddd, *J* = 8.0, 2.0, 1.1 Hz, 1H), 7.50 (dt, *J* = 7.7, 1.3 Hz, 1H), 7.40 (d, *J* = 16.0 Hz, 1H), 7.31 (t, *J* = 7.9 Hz, 1H), 6.70 (dd, *J* = 16.0, 7.5 Hz, 1H). ^13^C NMR (100 MHz, Chloroform-d) *δ* 193.21, 150.62, 136.07, 133.98, 131.23, 130.61, 129.69, 126.92, 123.25. MS (EI) *m/z*: 210, found 210.
Preparation of (*E*)-3-(2-chlorophenyl)acrylaldehyde (CAD-9) [23]: Under an argon atmosphere, 2-chlorobenzaldehyde (140 mg, 1 mmol) was dissolved in 3 mL tetrahydrofuran in an ice bath; 40% acetaldehyde aqueous solution (170 μL, 1.2 mmol) and 1 mL 1 mol/L NaOH aqueous solution were added to react for 3 h. The crude products were purified via column chromatography (dichloromethane: methanol = 10:1). The product compound 9 was obtained as a pale yellow oil, 135 mg, with a yield of 81.4%. ^1^H NMR (400 MHz, CDCl_3_) *δ* 9.79 (d, *J* = 7.6 Hz, 1H), 8.12 (dd, *J* = 8.1, 1.2 Hz, 1H), 8.05 (d, *J* = 15.9 Hz, 1H), 7.77–7.67 (m, 2H), 7.62 (ddd, *J* = 8.6, 6.8, 2.0 Hz, 1H), 6.64 (dd, *J* = 15.9, 7.6 Hz, 1H). ^13^C NMR (100 MHz, Chloroform-d) *δ* 193.13, 147.30, 133.83, 132.67, 131.16, 130.04, 129.09, 125.24. MS (EI) *m/z*: 166, found 166.
Preparation of (*E*)-3-(3-chlorophenyl)acrylaldehyde (CAD-10) [28]: Under an argon atmosphere, 3-chlorobenzaldehyde (140 mg, 1 mmol) was dissolved in 3 mL tetrahydrofuran under ice bath, 40% acetaldehyde aqueous solution (170 μL, 1.2 mmol) and 1 mL 1 mol/L NaOH aqueous solution were added to react for 3 h. The crude products were purified via column chromatography (dichloromethane: methanol = 10:1). The product compound 10 was obtained as a pale yellow oil, 137 mg, with a yield of 77.5%. ^1^H NMR (400 MHz, CDCl_3_) *δ* 9.81 (d, *J* = 7.6 Hz, 1H), 8.15 (dd, *J* = 8.1, 1.2 Hz, 1H), 8.35 (d, *J* = 15.9 Hz, 1H), 7.77–7.83 (m, 2H), 7.42 (ddd, *J* = 8.6, 6.8, 2.0 Hz, 1H), 6.64 (dd, *J* = 15.9, 7.6 Hz, 1H). MS (EI) *m/z*: 166, found 166.
Preparation of (*E*)-3-(4-chlorophenyl)acrylaldehyde (CAD-11) [23]: Under an argon atmosphere, 4-chlorobenzaldehyde (140 mg, 1 mmol) was dissolved in 3 mL tetrahydrofuran in an ice bath; 40% acetaldehyde aqueous solution (170 μL, 1.2 mmol) and 1 mL 1 mol/L NaOH aqueous solution were added to react for 8 h. The crude products were purified via column chromatography (petroleum ether: ethyl acetate = 8:1). The product compound 11 was obtained as a pale yellow solid, 128.5 mg, with a yield of 77.4%. ^1^H NMR (400 MHz, CDCl_3_) *δ* 9.71 (d, *J* = 8.1 Hz, 1H), 7.57 (d, *J* = 8.4 Hz, 2H), 7.39–7.44 (m, 3H), 6.70 (dd, *J* = 16.0, 7.5 Hz, 1H). MS (EI) *m/z*: 166, found 166.
Preparation of (*E*)-3-(2,3-dichlorophenyl)acrylaldehyde (CAD-12) [29]: Under an argon atmosphere, 2,3-dichlorobenzaldehyde (174 mg, 1 mmol) was dissolved in 3 mL tetrahydrofuran in an ice bath; 40% acetaldehyde aqueous solution (170 μL, 1.2 mmol) and 1 mL 1 mol/L NaOH aqueous solution were added to react for 3 h. The crude products were purified via column chromatography (petroleum ether: ethyl acetate = 8:1). The product compound 12 was obtained as a pale yellow solid, 95.6 mg, with a yield of 47.8%. ^1^H NMR (400 MHz, CDCl_3_) *δ* 9.72 (d, *J* = 7.6 Hz, 1H), 7.81–7.74 (m, 1H), 7.65 (dd, *J* = 7.2, 2.1 Hz, 1H), 7.35 (t, *J* = 7.4 Hz, 1H), 7.32–7.25 (m, 1H), 6.56 (dd, *J* = 15.8, 6.4 Hz, 1H). MS (EI) *m/z*: 200, found 200.
Preparation of (*E*)-3-(4-(trifluoromethyl)phenyl)acrylaldehyde (CAD-13) [23]: Under an argon atmosphere, 4-(trifluoromethyl) benzaldehyde (174 mg, 1 mmol) was dissolved in 3 mL tetrahydrofuran in an ice bath; 40% acetaldehyde aqueous solution (170 μL, 1.2 mmol) and 1 mL 1 mol/L NaOH aqueous solution were added to react for 3 h. The crude products were purified via column chromatography (petroleum ether: ethyl acetate = 6:1). The product compound 13 was obtained as a pale yellow solid, 176.8 mg, with a yield of 88.1%. ^1^H NMR (400 MHz, CDCl_3_) *δ* 9.85 (d, *J* = 7.5 MHz, 1H), 7.69 (m, 4H), 7.51 (d, *J* = 16.0 Hz, 1H), 6.77 (dd, *J* = 16.0, 7.5 Hz, 1 H). MS (EI) *m/z*: 200, found 200.
Preparation of (*E*)-3-(perfluorophenyl)acrylaldehyde (CAD-14) [29]: Under an argon atmosphere, pentafluorobenzaldehyde (196 mg, 1 mmol) was added to a solution of (triphenylphosphoranylidene) acetaldehyde (334.4 g, 1.1 mmol) in toluene (8 mL), and the mixture was stirred for 4 h at 80 °C. The crude products were purified via column chromatography (petroleum ether: ethyl acetate = 5:1). The product compound 14 was obtained as a colorless oil, 184 mg, with a yield of 83%.IR (KBr) *v* (cm^-1^):2965.07, 2932.59, 1696.10, 1650.75, 1613.97, 1532.60, 1499.77, 1422.04, 1158.05, 1121.47, 1000.29, 980.57, 967.62, 664.87. ^1^H NMR (400 MHz, CDCl_3_) *δ* 9.74 (d, *J* = 7.3 Hz, 1H), 7.48 (d, *J* = 16.5 Hz, 1H), 6.99 (dd, *J* = 16.5, 7.4 Hz, 1H). ^13^C NMR (100 MHz, Chloroform-d) *δ* 193.23, 146.77, 146.69, 144.26, 144.18, 139.14, 136.62, 135.27, 135.25, 135.16, 135.11, 130.89, 128.81, 109.65. ^19^F NMR (376 MHz, Chloroform-d) *δ* -138.57–138.88 (m), -149.20, -161.02 (dd, *J* = 20.1, 13.2 Hz). MS (EI) *m/z*: 222, found 222.
Preparation of (*E*)-3-(2-nitrophenyl)acrylaldehyde (CAD-15) [23]: Under an argon atmosphere, thiophene-3-carbaldehyde (151 mg, 1 mmol) was dissolved in 3 mL tetrahydrofuran in an ice bath; 40% acetaldehyde aqueous solution (170 μL, 1.2 mmol) and 1 mL 1 mol/L NaOH aqueous solution were added to react for 3 h. The crude products were purified via column chromatography (petroleum ether: ethyl acetate = 6:1). The product compound 15 was obtained as a pale yellow solid, 46 mg, with a yield of 26%. ^1^H NMR (400 MHz, CDCl_3_) *δ* 9.79 (d, *J* = 7.7 Hz, 1H), 8.12 (dd, *J* = 8.1, 1.3 Hz, 1H), 8.05 (d, *J* = 15.8 Hz, 1H), 7.74–7.67 (m, 2H), 7.62 (ddd, *J* = 8.7, 6.9, 2.0 Hz, 1H), 6.65 (dd, *J* = 15.9, 7.7 Hz, 1H). ^13^C NMR (100 MHz, Chloroform-d) *δ* 193.20, 147.38, 133.87, 132.67, 131.17, 129.11, 125.27. MS (EI) *m/z*: 177, found 177.
Preparation of (*E*)-3-(3-nitrophenyl)acrylaldehyde (CAD-16) [30]: Under an argon atmosphere, thiophene-3-carbaldehyde (151 mg, 1 mmol) was dissolved in 3 mL tetrahydrofuran in an ice bath; 40% acetaldehyde aqueous solution (170 μL, 1.2 mmol) and 1 mL 1 mol/L NaOH aqueous solution were added to react for 3 h. The crude products were purified via column chromatography (petroleum ether: ethyl acetate = 6:1). The product compound 16 was obtained as a pale yellow solid, 63.5 mg, with a yield of 35.9%. ^1^H NMR (400 MHz, CDCl_3_) *δ* 9.78 (d, *J* = 7.6 Hz, 1H), 8.43 (s, 1H), 8.30 (d, *J* = 8.3 Hz, 1H), 7.90 (d, *J* = 7.9 Hz, 1H), 7.65 (t, *J* = 8.1 Hz, 1H), 7.54 (d, *J* = 16.0 Hz, 1H), 6.83 (dd, *J* = 16.1, 7.5 Hz, 1H). MS (EI) *m/z*: 177, found 177.
Preparation of (*E*)-3-(p-tolyl)acrylaldehyde (CAD-17) [28]: Under an argon atmosphere, 4-methylbenzaldehyde (120 mg, 1 mmol) was dissolved in 3 mL tetrahydrofuran in an ice bath; 40% acetaldehyde aqueous solution (170 μL, 1.2 mmol) and 1 mL 1 mol/L NaOH aqueous solution were added to react for 9 h. The crude products were purified via column chromatography (dichloromethane: methanol = 10:1). The product compound 17 was obtained as a pale yellow oil, 52 mg, with a yield of 35.6%. ^1^H NMR (400 MHz, CDCl_3_) *δ* 9.69 (d, *J* = 7.7 Hz, 1H), 7.50–7.39 (m, 3H), 7.26 (d, *J* = 3.1 Hz, 2H), 6.69 (dd, *J* = 15.9, 7.7 Hz, 1H), 2.40 (s, 3H). ^13^C NMR (100 MHz, Chloroform-d) *δ* 193.83, 152.96, 129.88, 128.56, 127.77, 21.61. MS (EI) *m/z*: 146, found 146.
Preparation of (*E*)-3-(4-(dimethylamino)phenyl)acrylaldehyde (CAD-18) [31]: Under an argon atmosphere, 4-(dimethylamino) benzaldehyde (149 mg, 1 mmol) was dissolved in 3 mL tetrahydrofuran in an ice bath; 40% acetaldehyde aqueous solution (170 μL, 1.2 mmol) and 1 mL 1 mol/L NaOH aqueous solution were added to react for 3 h. The crude products were purified via column chromatography (petroleum ether: ethyl acetate = 6:1). The product compound 18 was obtained as a pale yellow solid, 152.1 mg, with a yield of 86.9%. ^1^H NMR (400 MHz, CDCl_3_) *δ* 9.59 (d, *J* = 7.9 Hz, 1H), 7.45 (d, *J* = 8.9 Hz, 2H), 7.38 (d, *J* = 15.6 Hz, 1H), 6.69 (d, *J* = 8.9 Hz, 2H), 6.54 (dd, *J* = 15.6, 7.9 Hz, 1H), 3.05 (s, 6H). ^13^C NMR (100 MHz, CDCl_3_) *δ* 193.74, 153.90, 152.36, 130.52, 123.89, 121.89, 111.85, 40.15. MS (EI) *m/z*: 175, found 175.
Preparation of (*E*)-3-(pyridin-3-yl)acrylaldehyde (CAD-19) [28]: Under an argon atmosphere, CA (132 mg, 1 mmol) was dissolved in 3 mL tetrahydrofuran in an ice bath; 40% acetaldehyde aqueous solution (170 μL, 1.2 mmol) and 1 mL 1 mol/L NaOH aqueous solution were added to react for 3 h. The crude products were purified via column chromatography (dichloromethane: methanol = 10:1). The product compound 19 was obtained as a pale yellow oil, 82 mg, with a yield of 51.8%. ^1^H NMR (400 MHz, CDCl_3_) *δ* 9.71 (d, *J* = 7.7 Hz, 1H), 7.60–7.55 (m, 2H), 7.51 (s, 1H), 7.47 (s, 1H), 7.44 (dd, *J* = 5.1, 1.9 Hz, 4H), 6.73 (dd, *J* = 16.0, 7.7 Hz, 1H). ^13^C NMR (100 MHz, CDCl_3_) *δ* 193.73, 152.81, 134.03, 131.31, 129.14, 128.64, 128.52. MS (EI) *m/z*: 158, found 158.
Preparation of (*E*)-3-(pyridin-3-yl)acrylaldehyde (CAD-20) [23]: Under an argon atmosphere, 3-Pyridinecarboxaldehyde (107 mg, 1 mmol) was dissolved in 3 mL tetrahydrofuran in an ice bath; 40% acetaldehyde aqueous solution (170 μL, 1.2 mmol) and 1 mL 1 mol/L NaOH aqueous solution were added to react for 3 h. The crude products were purified via column chromatography (petroleum ether: ethyl acetate = 1:1). The product compound 20 was obtained as a pale yellow solid, 113 mg, with a yield of 85.1%. ^1^H NMR (400 MHz, CDCl_3_) *δ* 9.75 (d, *J* = 7.5 Hz, 1H), 8.83–8.75 (m, 1H), 7.94–7.89 (m, 1H), 7.50 (d, *J* = 16.1 Hz, 1H), 7.40 (dd, *J* = 8.0, 4.8 Hz, 1H), 7.13–6.96 (m, 1H), 6.79 (dd, *J* = 16.1, 7.6 Hz, 1H). ^13^C NMR (100 MHz, CDCl_3_) *δ* 193.03, 151.91, 150.10, 148.50, 134.4, 130.22, 123.96. MS (EI) *m/z*: 133, found 133.
Preparation of (*E*)-3-(pyridin-4-yl)acrylaldehyde (CAD-21) [23]: Under an argon atmosphere, isonicotinaldehyde (107 mg, 1 mmol) was dissolved in 3 mL tetrahydrofuran in an ice bath; 40% acetaldehyde aqueous solution (170 μL, 1.2 mmol) and 1 mL 1 mol/L NaOH aqueous solution were added to react for 3 h. The crude products were purified via column chromatography (dichloromethane: methanol = 10:1). The product compound 21 was obtained as a pale yellow solid, 34 mg, with a yield of 25.6%.^1^H NMR (400 MHz, CDCl_3_) *δ* 9.63 (d, *J* = 7.9 Hz, 1H), 8.62 (dd, J = 18.8, 5.8 Hz, 2H), 7.36 (t, *J* = 7.0 Hz, 2H), 7.12 (dd, *J* = 15.2, 4.5 Hz, 1H), 6.74 (d, *J* = 15.2 Hz, 1H). MS (EI) *m/z*: 133, found 133.
Preparation of (*E*)-3-(pyridin-2-yl)acrylaldehyde (CAD-22) [23]: Under an argon atmosphere, picolinaldehyde (107 mg, 1 mmol) was dissolved in 3 mL tetrahydrofuran in an ice bath; 40% acetaldehyde aqueous solution (170 uL, 1.2 mmol) and 1 mL 1 mol/L NaOH aqueous solution were added to react for 3 h. The crude products were purified via column chromatography (dichloromethane: methanol = 10:1). The product compound 22 was obtained as a pale yellow solid, 28 mg, with a yield of 21.1%.^1^H NMR (400 MHz, CDCl_3_) *δ* 9.61 (d, *J* = 7.9 Hz, 1H), 8.72 (d, *J* = 4.9 Hz, 2H), 7.76 (t, *J* = 7.0 Hz, 2H), 7.12 (dd, *J* = 15.2, 4.5 Hz, 1H), 6.74 (d, *J* = 15.2 Hz, 1H). MS (EI) *m/z*: 133, found 133.
Preparation of (*E*)-3-(thiophen-3-yl)acrylaldehyde (CAD-23) [32]: Under an argon atmosphere, thiophene-3-carbaldehyde (112 mg, 1 mmol) was dissolved in 3 mL tetrahydrofuran in an ice bath; 40% acetaldehyde aqueous solution (170 uL, 1.2 mmol) and 1 mL 1 mol/L NaOH aqueous solution were added to react for 3 h. The crude products were purified via column chromatography (petroleum ether: ethyl acetate = 3:1). The product compound 23 was obtained as a light yellow liquid, 103 mg, with a yield of 75.3%. ^1^H NMR (400 MHz, CDCl_3_) *δ* 9.66 (d, *J* = 7.8 Hz, 1H), 7.63 (dd, *J* = 2.9, 1.3 Hz, 1H), 7.49 (d, *J* = 15.8 Hz, 1H), 7.40 (ddd, *J* = 5.1, 2.9, 0.6 Hz, 1H), 7.33 (dd, *J* = 5.1, 1.3 Hz, 1H), 6.54 (dd, *J* = 15.8, 7.8 Hz, 1H). MS (EI) *m/z*: 138, found 138.
Preparation of (*E*)-3-(thiophen-2-yl)acrylaldehyde (CAD-24) [32]: Under an argon atmosphere, thiophene-2-carbaldehyde (112 mg, 1 mmol) was dissolved in 3 mL tetrahydrofuran in an ice bath; 40% acetaldehyde aqueous solution (170 uL, 1.2 mmol) and 1 mL 1 mol/L NaOH aqueous solution were added to react for 3 h. The crude products were purified via column chromatography (petroleum ether: ethyl acetate = 5:1). The product compound 24 was obtained as a pale yellow liquid, 110 mg, with a yield of 79.7%. ^1^H NMR (400 MHz, CDCl_3_) *δ* 9.63 (d, *J* = 7.7 Hz, 1H), 7.59 (d, *J* = 15.7 Hz, 1H), 7.51 (dt, *J* = 5.0, 1.0 Hz, 1H), 7.39–7.34 (m, 1H), 7.12 (dd, *J* = 5.1, 3.7 Hz, 1H), 6.52 (dd, *J* = 15.7, 7.7 Hz, 1H). ^13^C NMR (100 MHz, CDCl_3_) *δ* 192.91, 144.42, 139.30, 132.07, 130.40, 128.54, 127.41. MS (EI) *m/z*: 138, found 138.
Preparation of (*E*)-3-(furan-3-yl)acrylaldehyde (CAD-25) [33]: Under an argon atmosphere, furan-3-carbaldehyde (96 mg, 1 mmol) was dissolved in 3 mL tetrahydrofuran in an ice bath; 40% acetaldehyde aqueous solution (170 μL, 1.2 mmol) and 1 mL 1 mol/L NaOH aqueous solution were added to react for 3 h. The crude products were purified via column chromatography (petroleum ether: ethyl acetate = 3:1). The product compound **25** was obtained as a colorless liquid, 72 mg, with a yield of 59.0%. ^1^H NMR (400 MHz, CDCl_3_) *δ* 9.67 (dd, *J* = 7.2, 1.9 Hz, 1H), 7.78 (d, *J* = 1.3 Hz, 1H), 7.46 (d, *J* = 7.8 Hz, 1H), 7.39 (m, 1H), 6.62 (dd, *J* = 7.8, 1.9 Hz, 1H), 6.46 (m, 1H). MS (EI) *m/z*: 122, found 122.

### 4.2. Biological Assay 

#### 4.2.1. Cell Culture and Cell Viability Detection

The HepG-2, HUVEC, L-02, HK-2, A375, A549, H8, U251, HeLa, Cal-27, and PANC-1 cells were maintained in a DMEM medium. The TSSCA, SK-MEL-1, and HK-2 cells were cultured in an RPMI-1640 medium, and the A875 cells were cultured in a MEM medium. All cells were supplemented with 10% fetal bovine serum, 100 units/mL penicillin, and streptomycin at 37 °C in 5% CO_2_. The Cell Counting Kit-8 (CCK-8, bs-0764P, Bioss, Beijing, China) method was used to assay the cytotoxicity. 

#### 4.2.2. Colony Formation Assays 

The A375 cells were plated onto six-well plates (500 cells/well), and after 24 h, CAD-14 (0–0.02 µM) was added to the medium and incubated for an additional 14 days. Cells were fixed in methanol and stained with Giemsa. The colonies were then scored and photographed. 

#### 4.2.3. Western Blotting Assay

The A375 cells were treated with CAD-14 (0–0.8 µM), alone or with indicated agents, for 24 h. Whole-cell extracts were loaded for SDS-PAGE gels depending on the target protein sizes, and were explored with protein-specific antibodies followed by HRP-conjugated secondary antibodies. Bands corresponding to the antibodies were detected via ECL chemiluminescence.

#### 4.2.4. Cell Cycle Analysis 

The A375 cells were incubated with CAD-14 (0–0.4 µM) for 24 h. The cells were collected, washed in PBS, and fixed in ice-cold 75% (*v*/*v*) ethanol overnight at –20 °C. The cells were re-suspended, and the DNA content was tested using flow cytometry. 

#### 4.2.5. Cellular Thermal Shift Assay (CETSA)

In A375 cell lysates, CAD-14 (0.4 μM) was added for 24 h at different temperatures. Western blotting was used to quantify the effects on ENO1 protein levels.

#### 4.2.6. Molecular Dynamics (MD) Assay 

The three-dimensional structure of ENO1 (PDB ID: 3B97) was obtained from the PDB database. Molecular docking was performed using Schrödinge software (Schrödinge_2018, Schrödinge Inc., New York, NY, USA). All crystalline water molecules were removed and the structure of the ENO1 protein was minimized. Multiple ligand structures were generated using different ionization states, tautomerism and stereoisomers at pH 6.8–7.2. All cysteine residues in the protein were involved in the reaction, and the reaction type was a Michael addition. The binding site was analyzed using Pymol software (Schrödinge_2018, Schrödinge Inc., New York, NY, USA).

#### 4.2.7. Building the Low ENO1-Expressing A375 Cells 

The recombinant lentiviral vectors (1348084) were purchased from Genechem (Shanghai, China). The formal transfection was conducted with HitransG P (a virus infection enhancer, MOI = 50), and subsequent experiments were performed after 72 h. The transfected cells were cultured with complete medium containing 1 μg/mL puromycin (01100205, Labgic Technology, Beijing, China) to maintain the transfection efficiency.

#### 4.2.8. Xenografts In Vivo 

The 8-week-old female BALB/c-nude mice (18–20 g, specific pathogen-free, SPF) were obtained from the Laboratory Animal Experimental Center of the Academy of Military Medical Sciences (SCXK2012-0004, Beijing, China), and were injected with A375 cells and siENO1-A375 cells (1 × 10^7^), respectively. One weeks after the tumor formation, four groups were set up: the “negative control (NC)” group (A375 cells, injected with PBS/day); “siENO1” group (siENO1-A375 cells, injected with PBS/day); “Dacarbazine” group (A375 cells, injected with 10 mg/kg/day dacarbazine); and the “CAD-14” group (A375 cells, injected with 5 mg/kg/day CAD-14). The tumor volume was measured every three days and calculated as length × width^2^/2. After two weeks, the mice were sacrificed. 

#### 4.2.9. Immunohistochemical Staining and Immunofluorescent Staining

Tumor tissues were isolated from the mice after sacrifice. Then, the tissues were fixed in 4% paraformaldehyde and embedded in paraffin. For immunohistochemistry staining, tissues were stained with Ki67 (GB111141, Servicebio, Wuhan, China). For immunofluorescent staining, tissues were stained with ENO1 (bs-3978R, Bioss, Beijing, China).

#### 4.2.10. Developmental Toxicity Test of *Zebrafish* Embryos 

The AB-wild-type adult *zebrafish* (three months old) were purchased from the Institute of Aquatic Biology, Chinese Academy of Sciences (Wuhan, China). During breeding, male and female fish were placed in separate spawning boxes at a 1:1 ratio. Then, early in the morning, they were mixed and laid eggs. During the exposure period, 96-well plates containing normal embryos were randomly selected and viewed daily under a light microscope to assess the developmental toxicity.

#### 4.2.11. Statistical Analysis 

The results are expressed herein as mean values ± SD, where significant differences between two groups were analyzed via a *t*-test, and the analysis of multiple groups was conducted with an analysis of variance (ANOVA) test. Differences of *p* < 0.05 were considered statistically significant.

## 5. Conclusions

This study demonstrated that CAD-14 could arrest the cell cycle and induce cell apoptosis, possibly via regulating the p38 pathway. CAD-14 bound to ENO1 and inhibited its stability. Meanwhile, CAD-14 could inhibit tumor growth by inhibiting ENO1 in vivo. Furthermore, an acute toxicity study demonstrated that CAD-14 has superior safety and tolerability compared with CA in vivo. These findings provide new understanding and indicate the potential of CAD-14 for the melanoma treatment. 

## Figures and Tables

**Table 1:** Mortalities and malformation rates after treating CAD-14 for 96 h.

	CAD-14 (μM)	Survival Number	Number of Deaths	Number of Deformities	Mortalities	Malformation Rates
	0	20	0	0	0.00%	0.00%
	8	20	0	0	0.00%	0.00%
96 h	16	20	0	0	0.00%	0.00%
	32	20	0	0	0.00%	0.00%
	64	20	0	0	0.00%	0.00%
	128	2	18	0	90.00%	0.00%
	D	20	0	0	0.00%	0.00%

## Data Availability

All data generated or analyzed during this study are included in this published article and its Supplementary Materials.

## Supplementary Materials

The following supporting information can be downloaded at: https://www.mdpi.com/article/10.3390/molecules28217309/s1, Figure S1: Original unedited bands for evaluating CAD-14 treatment (0–0.8 μM)-affected apoptotic proteins, as measured via Western blotting in Figure 3C; Figure S2: Original unedited bands for evaluating CAD-14 treatment (0–0.8 μM)-affected ENO1 proteins, as measured by Western blotting in Figure 4A; Figure S3: Original unedited bands for evaluating different temperature (37–92 °C)-affected ENO1 proteins, as measured by Western blotting in Figure 4B; Figure S4: Original unedited bands for evaluating CAD-14 treatment (0–0.8 μM)-affected P38/P-P38 proteins, as measured by Western blotting in Figure 4D; Figure S5: Original unedited bands for evaluated the effect of recombinant retrovirus on ENO1 expression after transfection via Western blotting in Figure 5B; Figure S6: The ^1^H NMR (400 MHz, CDCl3) of CAD-14.; Figure S7: The ^13^C NMR ^1^ (100 MHz, CDCl3) of CAD-14; Figure S8: The ^19^F NMR^1^ (376 MHz, CDCl3) of CAD-14; Figure S9: The GC of CAD-14; Figure S10: The MS of CAD-14; Figure S11: The IR of CAD-14; Figure S12: Developmental effects of CAD-14 on embryos; Table S1: Molecular docking of analogues.
