# Supplementary material for: Evaluating the Anti-Melanoma Effects and Toxicity of Cinnamaldehyde Analogues
Source: Molecules. 2023 Oct 28;28(21):7309. doi: 10.3390/molecules28217309 (PMC10647553; doi:10.3390/molecules28217309)
Supplement: Supplementary file 1 [file molecules-28-07309-s001.zip › molecules-2631059-supplementary.pdf]

Article

Evaluating the Anti−Melanoma Effects and Toxicity of Cinnamaldehyde Derivative Analogues

Rongsong Jiang [1,†], Fukui Shen [2,†], Miaomiao Zhang [1], Shulipan Mulati [1], Jinfeng Wang [1], Yicun Tao [1,*] and Weiyi Zhang [1,*]

1 School of Pharmacy, Xinjiang Medical University, Urumchi 830017, China; jrs@stu.xjmu.edu.cn (R.J.); zmm@stu.xjmu.edu.cn (M.Z.); slp@stu.xjmu.edu.cn (S.M.); wjf@xjmu.edu.cn (J.W.)
2 State Key Laboratory of Medicinal Chemical Biology, College of Pharmacy, Nankai University, Tianjin 300353, China; fukuishen@126.com
* Correspondence: taoyicun@xjmu.edu.cn (Y.T.); zwy@xjmu.edu.cn (W.Z.); Tel.: +86-0991-4362505 (W.Z.)
† These authors contributed equally to this work.

In this section, we have provided all the original blots. Each experiment was repeated three times. We have marked the molecular weight markers on the Western blotting images in the final figures of the manuscript. Image software was used for all sensitivity analyses, and we adopt the relative quantitative statistics and normalized the value.

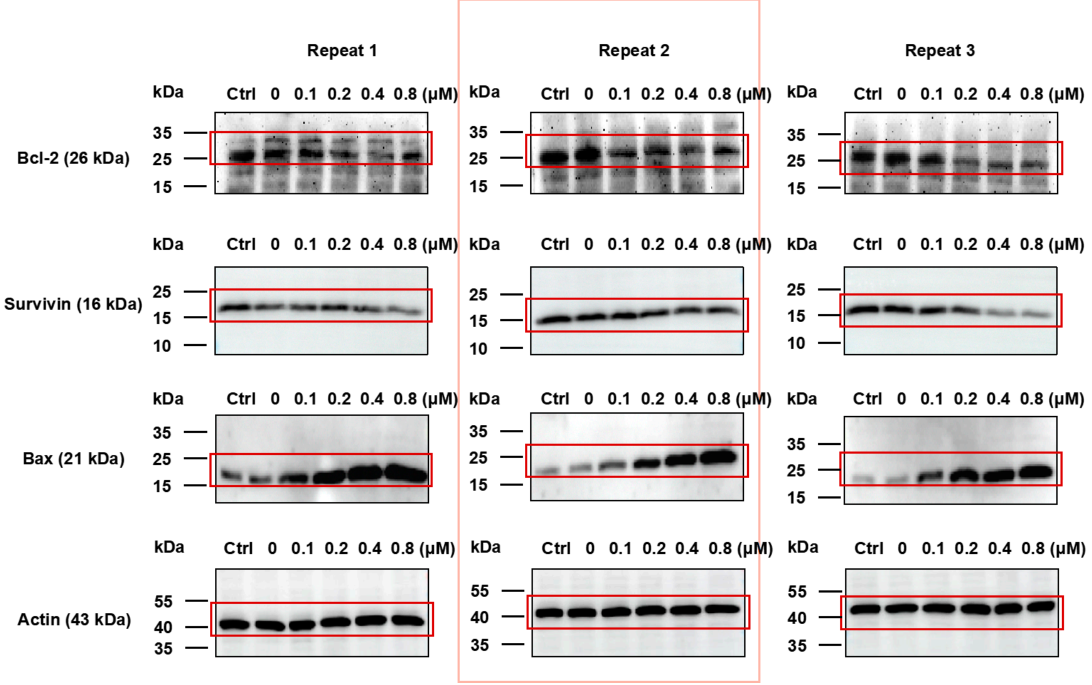

		Repeat 1			Repeat 2			Repeat 3				
Bax	CAD-14 (μM)	intden	Normalization to Actin (with same group)	Quadratic normalization to 0 (in each group)	intden	Normalization to Actin (with same group)	Quadratic normalization to 0 (in each group)	intden	Normalization to Actin (with same group)	Quadratic normalization to 0 (in each group)	The mean value of the three normalized groups	The standard deviation of the three normalized groups
	Ctrl	30288642	0.476804311	1	29001641	0.484371866	1	33250222	0.577604323	1	1	0
	0	32632932	0.522667459	1.096188617	26310087	0.431427668	0.890695143	33271314	0.586332552	1.015111087	1.000664949	0.103505605
	0.1	54286876	0.900524263	1.88866636	37435916	0.630620483	1.301934583	55798867	0.954700623	1.65286267	1.614487871	0.295242296
	0.2	68716771	1.11832141	2.345451547	47432839	0.792047415	1.635205243	64951628	1.075811506	1.862540606	1.947732465	0.36270607
	0.4	69335068	1.152197907	2.416500606	63858851	1.082488736	2.234829915	77841692	1.303109048	2.256058337	2.302462952	0.09932825
	0.8	70515572	1.14952296	2.410890447	87789328	1.454104538	3.002041696	95339562	1.586418656	2.746549141	2.719827095	0.296480186
Bcl-2												
	Ctrl	56352782	0.887106441	1	65375278	1.091867367	1	56337363	0.978661267	1	1	0
	0	48373937	0.77478428	0.873383671	60187635	0.986945085	0.903905652	58365543	1.028562255	1.050989029	0.942759451	0.09496383
	0.1	33749472	0.559844674	0.631090756	33253674	0.560169223	0.513037792	42336827	0.724369459	0.740163613	0.628097387	0.113592495
	0.2	22937474	0.37329269	0.420798083	26547392	0.443296114	0.405998134	15434393	0.255644055	0.261218119	0.362671445	0.088172233
	0.4	12747492	0.211835569	0.238793858	11028373	0.186944948	0.171215803	12973457	0.217182191	0.221917632	0.210642431	0.035171665
	0.8	15633735	0.254856294	0.287289419	10286355	0.170378745	0.156043444	11973538	0.199235697	0.203579832	0.215637565	0.066448613
Survivin												
	Ctrl	46353746	0.729701449	1	54363527	0.907954244	1	76353637	1.326372822	1	1	0
	0	45363462	0.726566813	0.995704222	55635472	0.912299605	1.004785881	75334372	1.327599943	1.00092517	1.000471758	0.004557776
	0.1	37448209	0.621200248	0.851307407	53826354	0.906722875	0.998643798	55499736	0.949582588	0.715924341	0.855291849	0.141401838
	0.2	25343622	0.412451206	0.565232818	38252728	0.638755237	0.703510382	53423211	0.884863195	0.667130071	0.64529109	0.071678986
	0.4	23223169	0.385918519	0.5288718	24235118	0.410816071	0.452463407	45343622	0.759075022	0.57229386	0.517876356	0.0606672
	0.8	21928364	0.357469381	0.489884434	16028364	0.265486903	0.292401192	43524252	0.724229102	0.546022272	0.442769299	0.133213325
Actin												
	Ctrl	63524262	1		59874743	1		57565743	1			
	0	62435362	1		60983773	1		56744784	1			
	0.1	60283635	1		59363622	1		58446455	1			
	0.2	61446352	1		59886363	1		60374543	1			
	0.4	60176353	1		58992624	1		59735363	1			
	0.8	61343335	1		60373464	1		60097353	1			

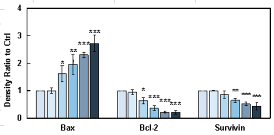

Supplement Figure S1 Original unedited bands for evaluating CAD−14 treatment (0−0.8 μM) affected apoptotic proteins measured by western blotting in Figure 3C. Image software was used for sensitivity analysis, and the relative quantitative statistics and normalized the intensity value against "Ctrl" group were adopted. Western blotting was performed 3 times. The "Repeat 2" was used in Figure 3C of the manuscript.

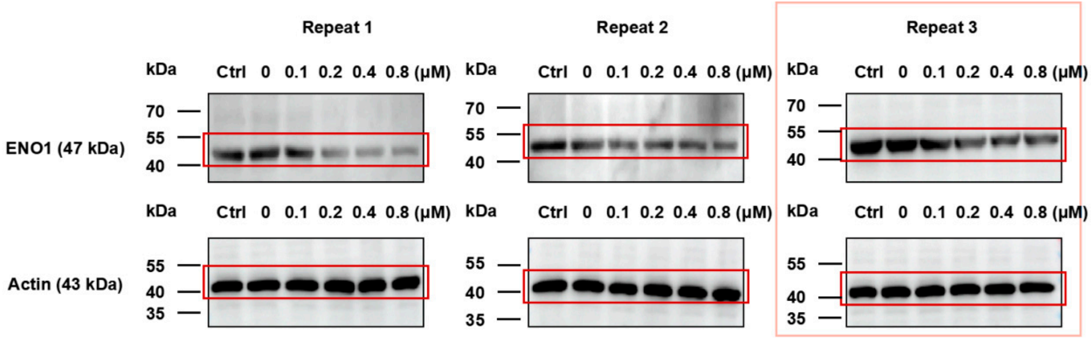

		Repeat 1			Repeat 2			Repeat 3				
ENO1	CAD-14 (μM)	intden	Normalization to Actin (with same group)	Quadratic normalization to 0 (in each group)	intden	Normalization to Actin (with same group)	Quadratic normalization to 0 (in each group)	intden	Normalization to Actin (with same group)	Quadratic normalization to 0 (in each group)	The mean value of the three normalized groups	The standard deviation of the three normalized groups
	Ctrl	47435111	0.773958302	1	43402270	0.842572479	1	60305643	1.121186399	1	1	0
	0	48567546	0.827996662	1.069820764	42765756	0.773541916	0.918071661	59739447	1.082659969	0.965637801	0.984510076	0.07761488
	0.1	36030520	0.669233604	0.864689483	37577782	0.647914979	0.768972397	50711303	0.892003523	0.795588962	0.809750281	0.049404933
	0.2	25229748	0.426420003	0.550959919	25611181	0.445160548	0.528335021	40268223	0.68500557	0.61096493	0.563419957	0.042700877
	0.4	25236298	0.425267436	0.549470734	24143491	0.432874814	0.513753801	40438961	0.754159533	0.672644204	0.578622913	0.083360226
	0.8	24919081	0.448198928	0.579099581	22503069	0.410871825	0.487639741	40914783	0.724665411	0.646338032	0.571025785	0.079656617
Actin												
	Ctrl	61288975	1		51511616	1		53787348	1			
	0	58656693	1		55285635	1		55178402	1			
	0.1	53838480	1		57998014	1		56851012	1			
	0.2	59166427	1		57532459	1		58785249	1			
	0.4	59342183	1		55774765	1		53621229	1			
	0.8	55598261	1		54769073	1		56460240	1			

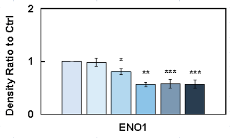

Supplement Figure S2 Original unedited bands for evaluating CAD−14 treatment (0−0.8 μM) affected ENO1 protein measured by western blotting in Figure 4A. Image software was used for sensitivity analysis, and the relative quantitative statistics and normalized the intensity value against "Ctrl" group were adopted. Western blotting was performed 3 times. The "Repeat 3" was used in Figure 4A of the manuscript.

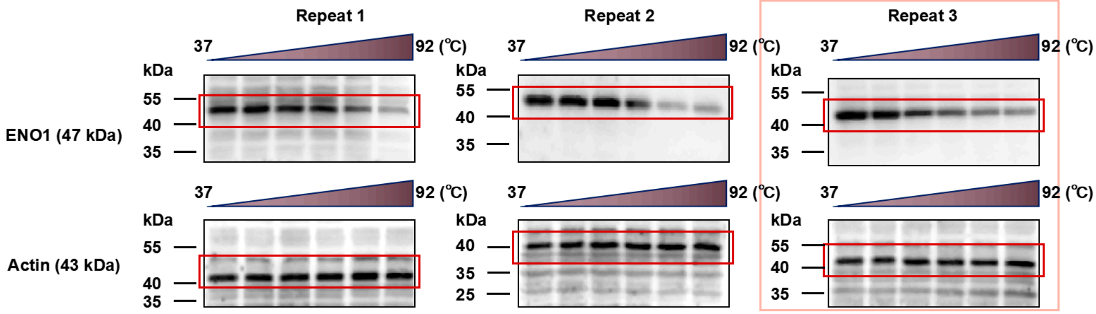

		Repeat 1			Repeat 2			Repeat 3				
CAD-14	Temp(℃)	intden	Normalization to Actin (with same group)	Quadratic normalization to 0 (in each group)	intden	Normalization to Actin (with same group)	Quadratic normalization to 0 (in each group)	intden	Normalization to Actin (with same group)	Quadratic normalization to 0 (in each group)	The mean value of the three normalized groups	The standard deviation of the three normalized groups
	37	45230	0.902380145	1	62516	1.416857421	1	61026	1.148097979	1	1	0
ENO1	48	46251	0.88676496	0.982695558	61025	1.348082531	0.951459554	60892	1.166871072	1.016351472	0.983502195	0.032453478
	59	31156	0.606513656	0.672126553	58620	1.165175909	0.822366381	29216	0.558121764	0.486127294	0.660206743	0.168436167
	70	19127	0.365500373	0.405040353	14306	0.308465221	0.217710841	11745	0.221203104	0.192669187	0.271806794	0.116061006
	81	9610	0.171650055	0.190219229	12036	0.259000237	0.182799083	10293	0.197285953	0.171837209	0.181618507	0.009247702
	92	5074	0.100082844	0.110909847	9851	0.193989878	0.136915596	8456	0.155968718	0.135849658	0.1278917	0.014716371
Actin	Temp(℃)											
	37	50123	1		44123	1		53154	1			
	48	52157	1		45268	1		52184	1			
	59	51369	1		50310	1		52347	1			
	70	52331	1		46378	1		53096	1			
	81	55986	1		46471	1		52173	1			
	92	50698	1		50781	1		54216	1			

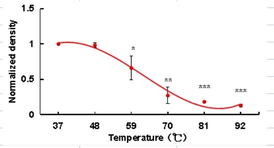

Supplement Figure S3 Original unedited bands for evaluating different temperature (37−92℃) affected ENO1 protein measured by western blotting in Figure 4B. Image software was used for sensitivity analysis, and the relative quantitative statistics and normalized the intensity value against "37℃" group were adopted. Western blotting was performed 3 times. The "Repeat 3" was used in Figure 4B of the manuscript.

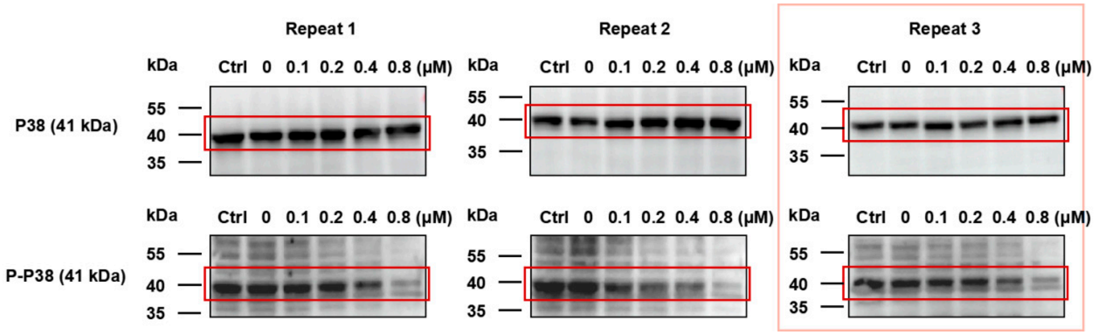

		Repeat 1			Repeat 2			Repeat 3				
P-p38	CAD-14 (μM)	intden	Normalization to p38 (with same group)	Quadratic normalization to 0 (in each group)	intden	Normalization to Actin (with same group)	Quadratic normalization to 0 (in each group)	intden	Normalization to Actin (with same group)	Quadratic normalization to 0 (in each group)	The mean value of the three normalized groups	The standard deviation of the three normalized groups
	Ctrl	92855999	1.532367838	1	60667766	1.312613669	1	86032286	2.88789285	1	1	0
	0	88181409	1.427411506	0.93150709	62388920	1.545829088	1.177672551	89090229	3.077332203	1.065597777	1.058259139	0.123246705
	0.1	74725115	1.330898939	0.868524453	56162373	1.119510288	0.852886355	82432538	2.415499546	0.836422842	0.852611217	0.016052574
	0.2	68084808	1.234112566	0.805363135	60956687	1.171676191	0.892628363	83179822	3.043978558	1.054048303	0.9173466	0.126171794
	0.4	65027341	1.274063998	0.831434833	52660803	0.844919616	0.643692531	73694385	2.161043781	0.748311621	0.741146329	0.094076028
	0.8	44234678	0.747074086	0.487529213	35917727	0.611234174	0.465661899	61042186	1.947962915	0.674527421	0.542572844	0.114797877
p38	Ctrl	60596416	1		46219057	1		29790678	1			
	0	61777146	1		40359520	1		28950475	1			
	0.1	56146348	1		50166911	1		34126497	1			
	0.2	55169042	1		52025199	1		27326021	1			
	0.4	51039305	1		62326406	1		34101292	1			
	0.8	59210564	1		58762629	1		31336421	1			

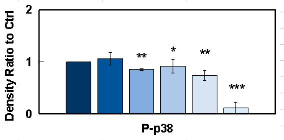

Supplement Figure S4 Original unedited bands for evaluating CAD−14 treatment (0−0.8 μM) affected P38/P−P38 proteins measured by western blotting in Figure 4D. Image software was

used for sensitivity analysis, and the relative quantitative statistics and normalized the intensity value against “Ctrl” group were adopted. Western blotting was performed 3 times. The “Repeat 3” was used in Figure 4D of the manuscript.

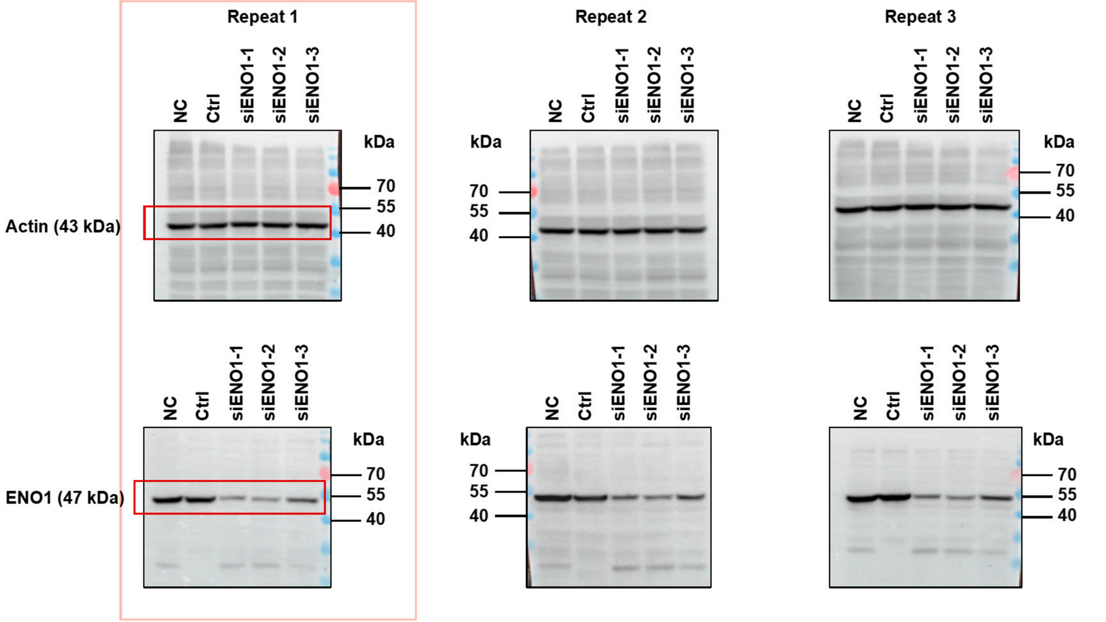

		Repeat 1			Repeat 2			Repeat 3				
ENO1	viral transdu	intden	Normalization to Actin (with same group)	Quadratic normalization to 0 (in each group)	intden	Normalization to Actin (with same group)	Quadratic normalization to 0 (in each group)	intden	Normalization to Actin (with same group)	Quadratic normalization to 0 (in each group)	The mean value of the three normalized groups	The standard deviation of the three normalized groups
	NC	35984.2	1.086256839	1.108403758	43450.125	1.069144041	1.016986501	41598.882	1.146468419	0.966279961	1.03055674	0.072027127
	Ctrl	34662.4	0.980019087	1	37142.004	1.051286364	0.983297221	37157.761	1.186476451	1.034896759	1.00606466	0.026328942
	siENO1-1	13306.7	0.40917765	0.417520083	20354.296	0.604460416	0.56536855	11015.497	0.353938387	0.308720573	0.430536402	0.128818145
	siENO1-2	13609.6	0.371939721	0.379522936	17393.175	0.463873442	0.433873664	11629.468	0.343194058	0.299348898	0.370915166	0.067674208
	siENO1-3	22859.5	0.602174427	0.614451734	21290.054	0.704410931	0.658855032	14905.539	0.436950998	0.381127811	0.551478192	0.149188983
Actin												
	NC	33126.8	1		40640.104	1		36284.368	1			
	Ctrl	35369.1	1		35330.054	1		31317.74	1			
	siENO1-1	32520.6	1		33673.497	1		31122.64	1			
	siENO1-2	36591	1		37495.518	1		33885.983	1			
	siENO1-3	37961.5	1		30223.912	1		34112.61	1			

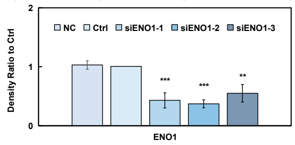

Supplement Figure S5 Original unedited bands for evaluated the effect of recombinant retrovirus on ENO1 expression after transfecting by western blotting in Figure 5B. Image software was used for sensitivity analysis, and the relative quantitative statistics and normalized the intensity value against “NC” group were adopted. Western blotting was performed 3 times. The “Repeat 1” was used in Figure 5B of the manuscript.

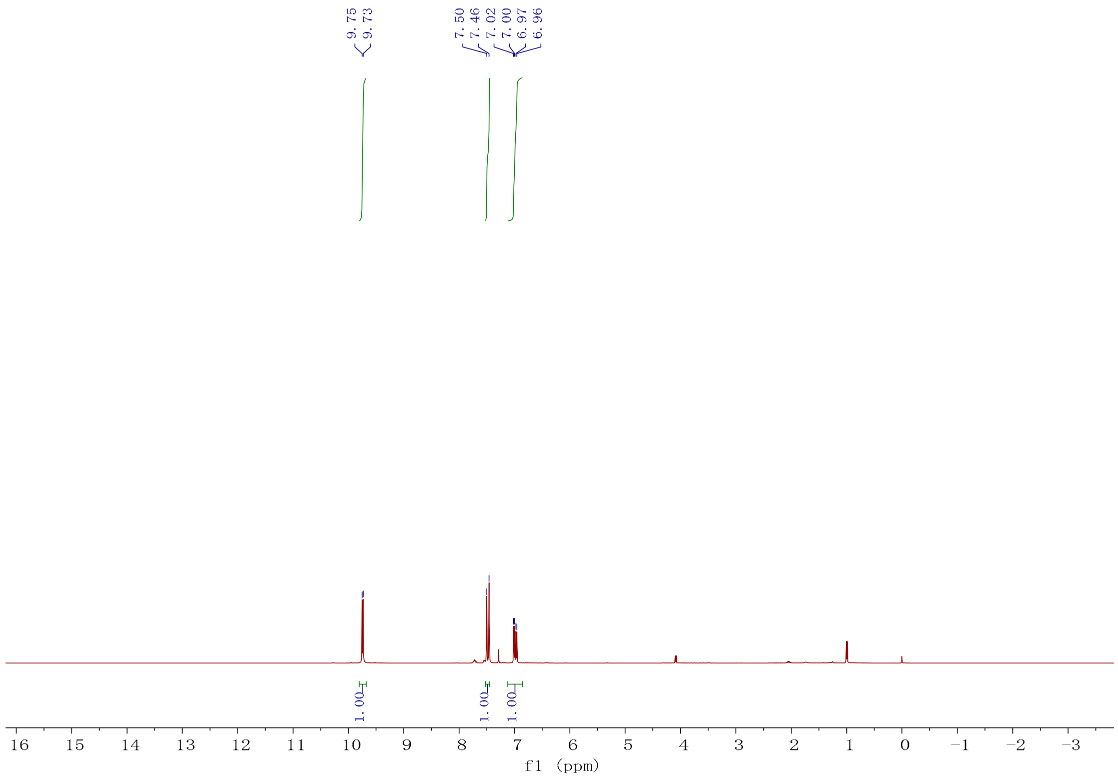

Supplement Figure S6 The ^{1}H NMR (400 MHz, $CDCl_3$) of CAD−14.

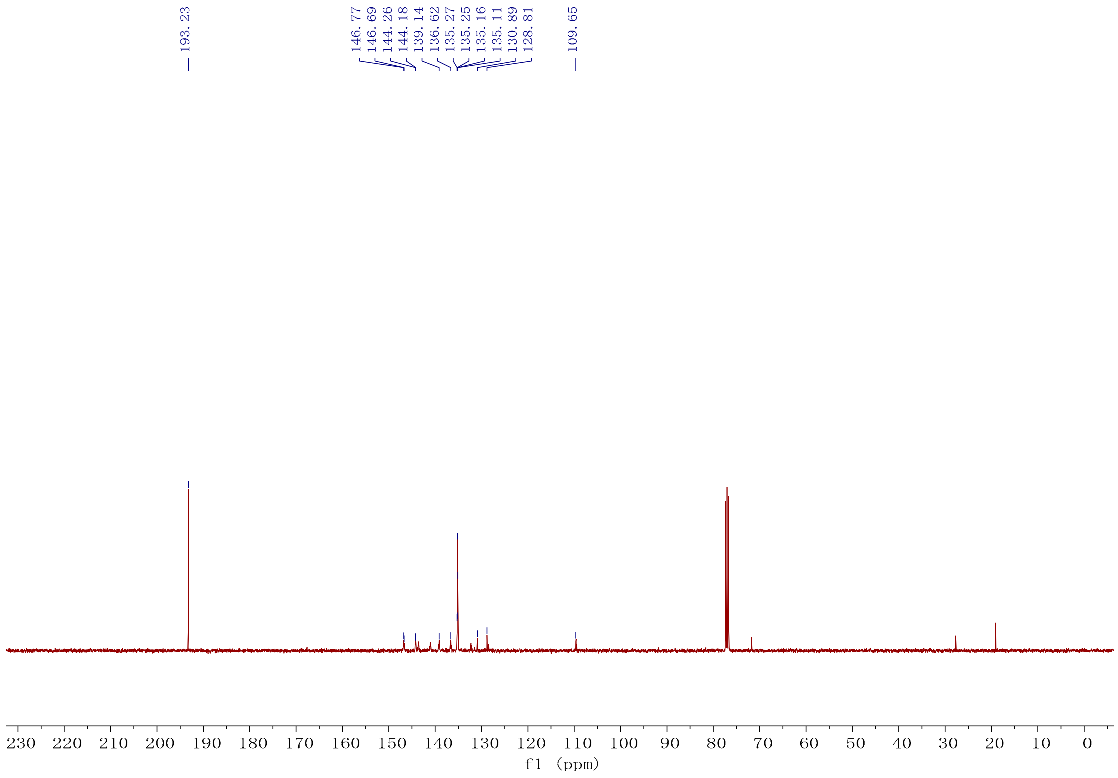

Supplement Figure S7 The ^{13}C NMR [1] (100 MHz, $CDCl_3$) of CAD−14.

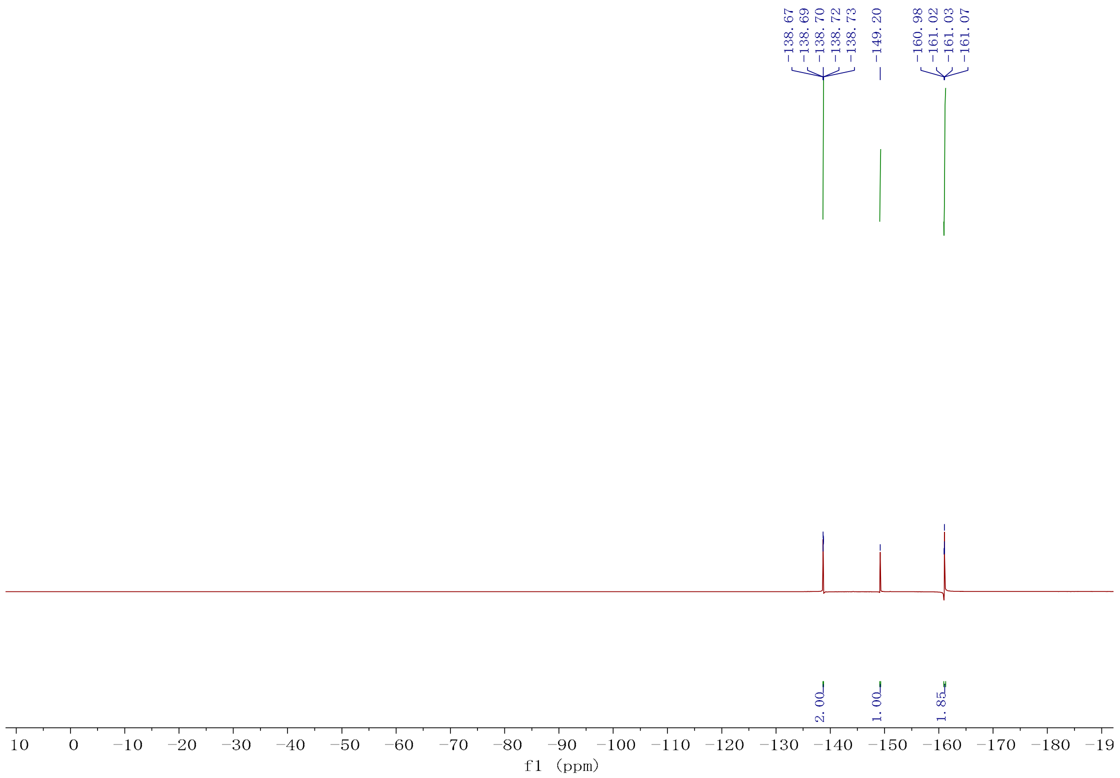

Supplement Figure S8 The ^{19}F NMR [1] (376 MHz, $CDCl_3$) of CAD−14.

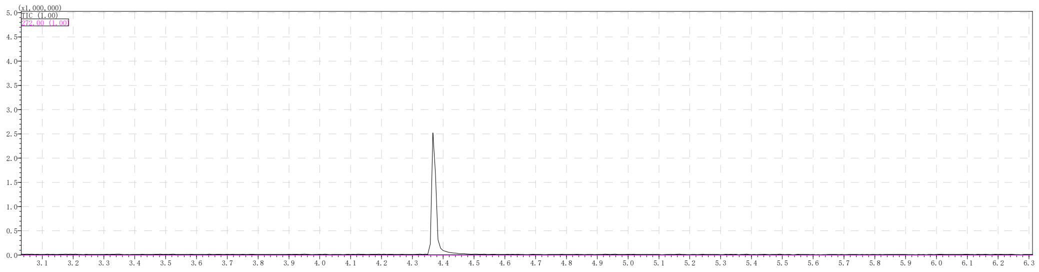

Supplement Figure S9 The GC of CAD−14.

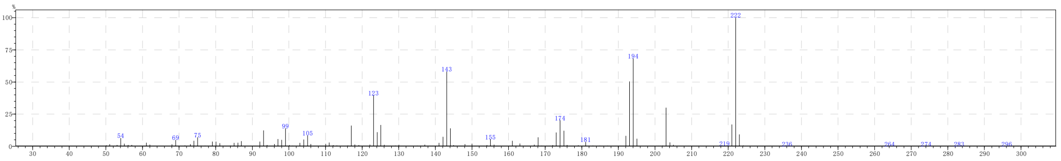

Supplement Figure S10 The MS of CAD−14.

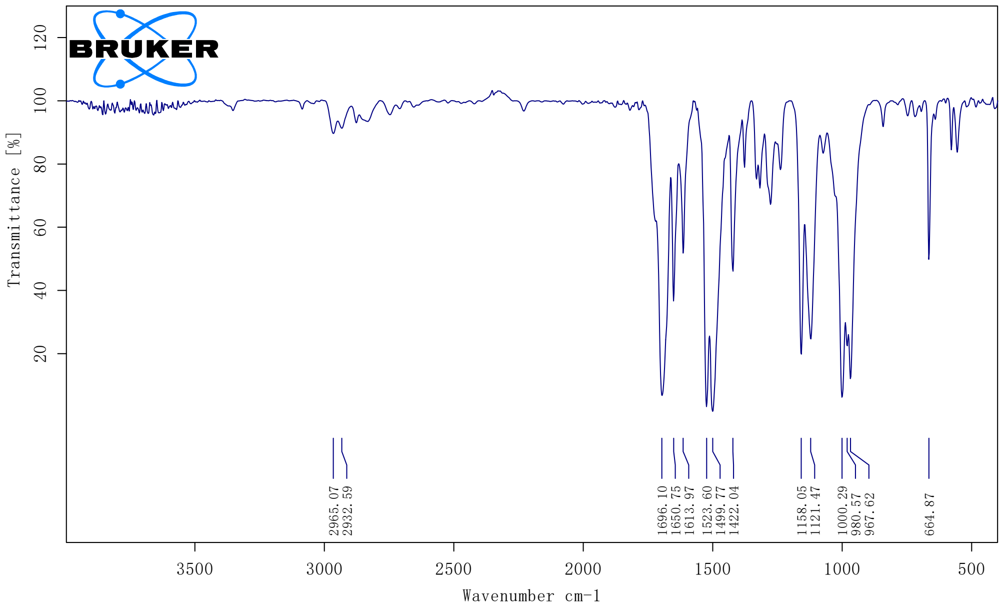

Supplement Figure S11 The IR of CAD−14.

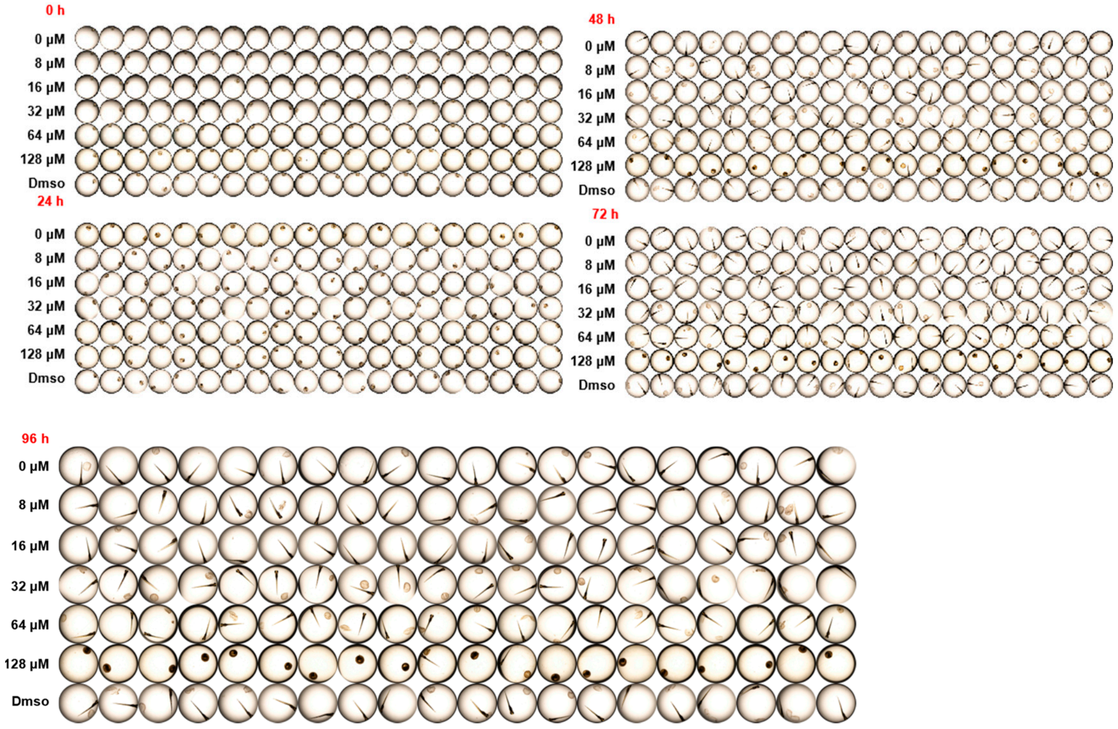

Supplement Figure S12 Developmental effects of CAD−14 on embryos. The embryo morphology after CAD−14 treatment for 0−96 h (n = 20).

Supplement Table S1 Molecular docking of Analogues

ID	Structure	Molecular docking figure	Score （kcal/mol）
CAD−1	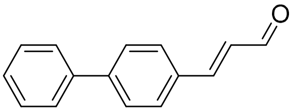	Unsuccessful	
CAD−2	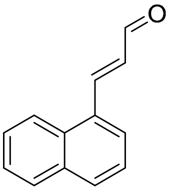	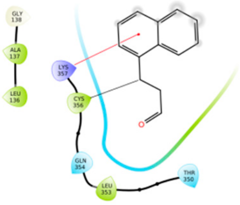	−1.525
CAD−3	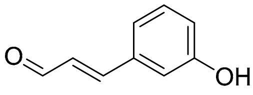	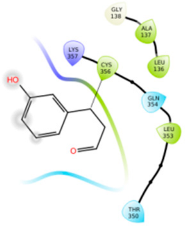	−2.656
CAD−4	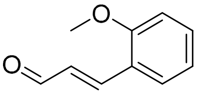	Unsuccessful	
CAD−5	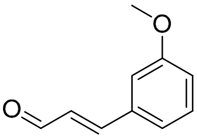	Unsuccessful	
CAD−6	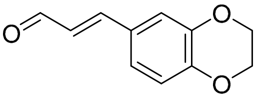	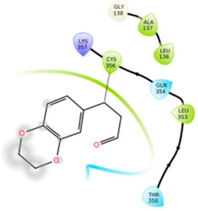	−2.738
CAD−7	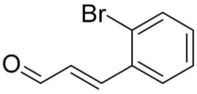	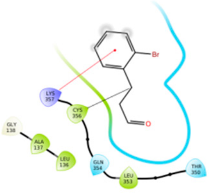	−1.232
CAD−8	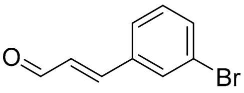	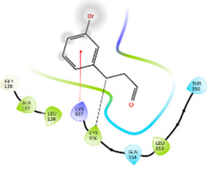	

**CAD−9**	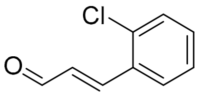	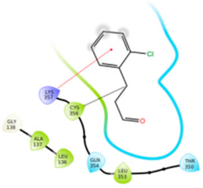	−2.714
**CAD−10**	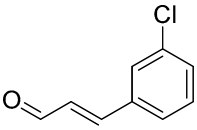	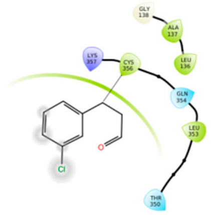	−2.586
**CAD−11**	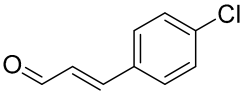	**Unsuccessful**	
**CAD−12**	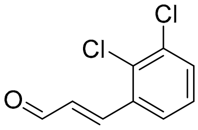	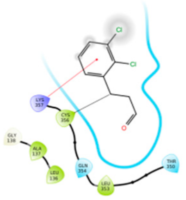	−1.124
**CAD−13**	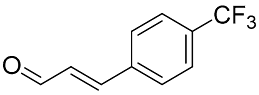	**Unsuccessful**	
**CAD−14**	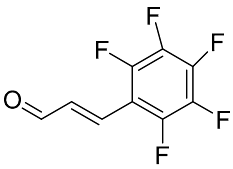	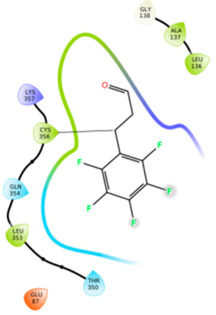	−2.397
**CAD−15**	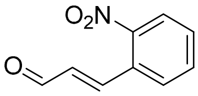	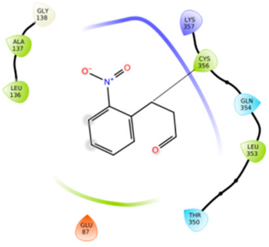	-2.188
**CAD−16**	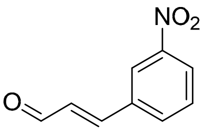	**Unsuccessful**	
**CAD−17**	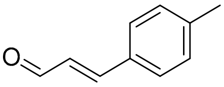	**Unsuccessful**	

CAD−18	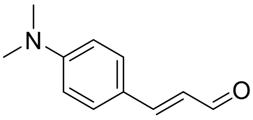	**Unsuccessful**	
CAD−19	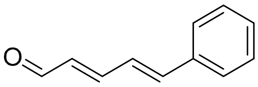	**Unsuccessful**	
CAD−20		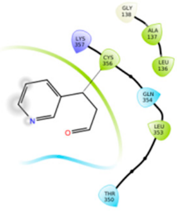	−2.391
CAD−21	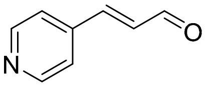	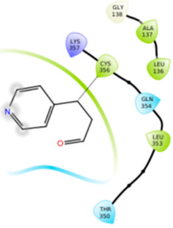	−2.567
CAD−22	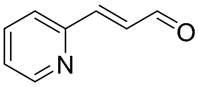	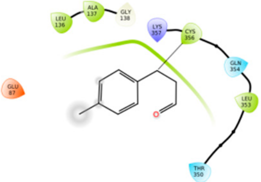	−1.642
CAD−23		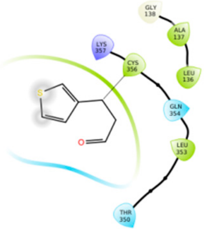	−2.711
CAD−24	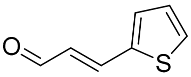	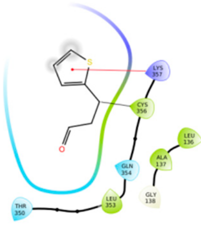	−1.905
CAD−25	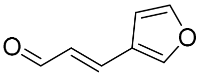	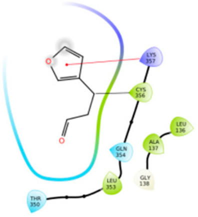	−1.879
